# Wernicke Encephalopathy Secondary to Cannabinoid Hyperemesis Syndrome in a 15-Year-Old Male Patient: A Case Report

**DOI:** 10.7759/cureus.115093

**Published:** 2026-08-24

**Authors:** Justin Abes, Melissa Bou Jaoude, Trinity Landrum, Janelle McGill

**Affiliations:** 1 Pediatrics, Medical College of Georgia at Augusta University, Augusta, USA

**Keywords:** adolescent cannabis use, cannabinoid hyperemesis syndrome, nystagmus, pediatric, thiamine deficiency, wernicke encephalopathy

## Abstract

The use of cannabis and high-potency tetrahydrocannabinol (THC) products has risen substantially among adolescents, and cannabinoid hyperemesis syndrome (CHS) is now an increasingly recognized complication of chronic use. CHS is characterized by recurrent episodes of intractable nausea, vomiting, and abdominal pain that resolve with cannabis cessation. Wernicke encephalopathy (WE), a neurologic syndrome caused by thiamine deficiency, is classically associated with chronic alcohol use but is increasingly reported in nonalcoholic and pediatric patients, in whom atypical presentations complicate timely diagnosis.

We report the case of a 15-year-old male patient with no significant past medical history and chronic cannabis use who developed WE in the setting of CHS. He initially presented with several days of intractable nausea, vomiting, and headache and was diagnosed with CHS. Symptoms persisted, and he returned weeks later with a 25- to 30-pound weight loss along with headache and dizziness. Repeat laboratory studies revealed worsening transaminitis and progressive hyponatremia, which was treated with fluid restriction and oral sodium supplementation. He was admitted for management of presumed CHS, and on admission demonstrated slowed speech, mild confusion, and episodes of unresponsiveness. Nephrology and gastroenterology were consulted.

Several days into admission, he showed little cognitive improvement, remained bedbound, and developed an ataxic gait once movement was initiated. This was followed by new-onset horizontal nystagmus with blurred vision. Brain magnetic resonance imaging and electroencephalography were unremarkable, and a workup for encephalopathy was unrevealing. Given the constellation of nystagmus, ataxia, altered mentation, and protracted vomiting with significant weight loss, WE was suspected; a vitamin panel was ordered, and empiric thiamine was initiated. The vitamin panel subsequently confirmed thiamine deficiency. Within one day of thiamine supplementation, the patient's nystagmus, gait, and cognitive function improved, consistent with a clinical diagnosis of WE likely secondary to CHS-induced thiamine deficiency. At follow-up, he reported cessation of cannabis, demonstrated adequate weight gain, and full neurologic recovery.

To our knowledge, this is among the few reported cases of WE associated with CHS and highlights that normal MRI findings do not exclude the diagnosis. As adolescent THC use continues to rise, clinicians should maintain a low threshold for empiric thiamine supplementation in adolescents with prolonged CHS and any new neurologic findings, regardless of imaging results.

## Introduction

The use of cannabis and higah-potency tetrahydrocannabinol (THC) products has risen substantially among adolescents in the United States over the past decade, mirroring trends in legalization and increased product potency [1]. Cannabinoid hyperemesis syndrome (CHS) is an increasingly recognized consequence of chronic cannabis use, characteristically developing after prolonged, frequent consumption over several months [2,3]. The diagnosis is clinical, based on recurrent episodes of nausea, vomiting, and abdominal cramping in a chronic cannabis user without an alternative explanation. Episodes are often relieved temporarily by hot-water bathing, and durable resolution occurs only with sustained cannabis cessation [2,3]. Although the first descriptions of CHS emphasized adult populations [4], the syndrome is now increasingly reported in adolescents and warrants further study in these populations [1].

Wernicke encephalopathy (WE) is a neurologic syndrome caused by a thiamine (vitamin B1) deficiency and is classically associated with chronic alcohol use disorder. It is classically characterized by the triad of clinical manifestations: nystagmus, ataxia, and alternations of consciousness, although recent meta-analytic data indicate that the complete triad is present in only 32.7% of patients overall [5]. Non-alcoholic causes such as hyperemesis gravidarum, anorexia nervosa, and severe malnutrition account for a growing share of reported cases [5]. In pediatric populations, a recent 30-year systematic review identified 88 cases of WE attributable to a range of etiologies, but CHS was not represented among them [6]. To our knowledge, only one pediatric case of WE associated with CHS has been previously reported [7]. In that report, brain magnetic resonance imaging (MRI) demonstrated typical WE findings. However, there are limited studies of WE in a pediatric population associated with CHS and negative MRI findings.

Here we report the case of a 15-year-old male patient diagnosed with WE likely secondary to nutritional depletion in the setting of CHS and negative MRI findings.

## Case presentation

A 15-year-old male patient with no significant past medical history initially presented to our emergency department (ED) with several days of persistent nausea, vomiting, and headache. The patient reported cannabis use over the past three months but declined to specify beyond that. There was no reported history of alcohol or other illicit substance use, and he had not experienced similar symptoms previously. A urine drug screen (UDS) was positive for THC. A diagnosis of possible cannabinoid hyperemesis syndrome was given in the ED due to his protracted vomiting and positive UDS. He received intravenous fluids, antiemetics (ondansetron and promethazine as needed), precautions that included stopping cannabis use, and was discharged following symptomatic improvement.

Symptoms persisted, prompting outpatient evaluation by his primary care physician three weeks later. The patient reported continued cannabis use at this point. A comprehensive metabolic panel (CMP) was ordered at that time and demonstrated elevated transaminases (aspartate aminotransferase (AST) 111 U/L, alanine aminotransferase (ALT) 506 U/L) and mild hyponatremia (serum sodium 130 mmol/L). Non-contrast head computed tomography (CT) was also ordered and was read as "showed increased attenuation of the intracranial vasculature that could represent hemoconcentration related to dehydration, minimal mucosal changes of the right maxillary sinus that are probably related to previous sinusitis, no mass or acute intracranial abnormality."

The patient returned to the ED with his mother days after his outpatient evaluation, approximately one month after his initial ED visit. His mother reported that he had lost 25 to 30 pounds since his initial visit and had developed new complaints of headache and dizziness over the preceding several days. Repeat laboratory studies revealed worsening transaminitis (peak ALT 714 U/L, AST 178 U/L), progressive hyponatremia (sodium 125 mmol/L), and a urinalysis notable for elevated specific gravity, glucosuria, and ketonuria (Table 1). Repeat UDS remained positive for THC. CT of the abdomen and pelvis at that time demonstrated nonspecific findings consistent with gastroenteritis, without evidence of appendicitis, obstruction, or hydronephrosis.

**Table 1:** Laboratory findings over the patient’s clinical course, with reference ranges. AST = aspartate aminotransferase; ALT = alanine aminotransferase; Na = sodium; K = potassium; UDS = urine drug screen; THC = tetrahydrocannabinol; PCP = primary care physician; ED = emergency department; U/L = units per liter; mmol/L = millimoles per liter; nmol/L = nanomoles per liter. Reference ranges reflect values within our institution and may vary by laboratory.

Laboratory test (units)	Reference range	Initial ED evaluation (1 month prior)	Outpatient follow-up (PCP)	Repeat ED evaluation/admission
Aspartate aminotransferase, AST (U/L)	10–40	21	111	178
Alanine aminotransferase, ALT (U/L)	7–45	28	506	714 (peak)
Serum sodium, Na (mmol/L)	135–145	136	130	125
Serum potassium, K (mmol/L)	3.5–5.1	3	4.2	3.3 (nadir 2.9)
Serum osmolality (mOsm/kg)	275–295	—	—	260
Urine sodium (mmol/L)	—	—	—	103
Urine specific gravity	1.005–1.030	1.02	—	1.048
Urine glucose	Negative	Negative	—	100
Urine ketones	Negative	Trace	—	20
Urine drug screen, UDS (THC)	Negative	Positive	—	Positive
Whole-blood thiamine, vitamin B1 (nmol/L)	70–180	—	—	39 (inpatient day 4)

The patient was admitted to the pediatric service for further management of his symptoms and abnormal lab values. On initial assessment by our pediatric service, the patient demonstrated slowed speech, mild confusion, and momentary episodes of unresponsiveness to verbal prompts. Initial treatment consisted of intravenous fluids and antiemetic therapy, which had improved his headache and dizziness. Persistent hyponatremia (125 mmol/L) the following day prompted nephrology consultation.

On day 2 of admission, nephrology recommended obtaining serum osmolality, urine sodium level, and a renal ultrasound. Laboratory results revealed hypotonic hyponatremia (serum osmolality, 260 mOsm/kg), an elevated urine sodium level (103 mmol/L), clinical euvolemia, and a "normal renal ultrasound." Several diagnoses were considered as causes for his hyponatremia, such as hypovolemic hyponatremia from gastrointestinal losses, but this was later excluded given his elevated urine sodium and consistent hyponatremia despite intravenous fluids. A clinical diagnosis of syndrome of inappropriate antidiuretic hormone secretion (SIADH) was suggested, presumed secondary to physiologic stress and inflammation from protracted vomiting. Treatment with fluid restriction and oral sodium tablets was trialed. On day 3 of admission, there was improvement in his hyponatremia (125 mmol/L to 129 mmol/L) but little improvement in his cognitive status and now new-onset blurred vision. Serum sodium subsequently normalized by day 4 of admission (133 mmol/L) following fluid restriction and oral sodium supplementation.

Gastroenterology was also consulted early in admission in the setting of persistent vomiting, weight loss, and transaminitis. Abdominal ultrasound and barium swallow studies were nonspecific. Pantoprazole was initiated and subsequently dose-escalated for presumed gastroesophageal reflux, and ondansetron was continued as needed. Hepatic transaminases trended downward during admission as fluids and electrolytes normalized.

**Figure 1:**
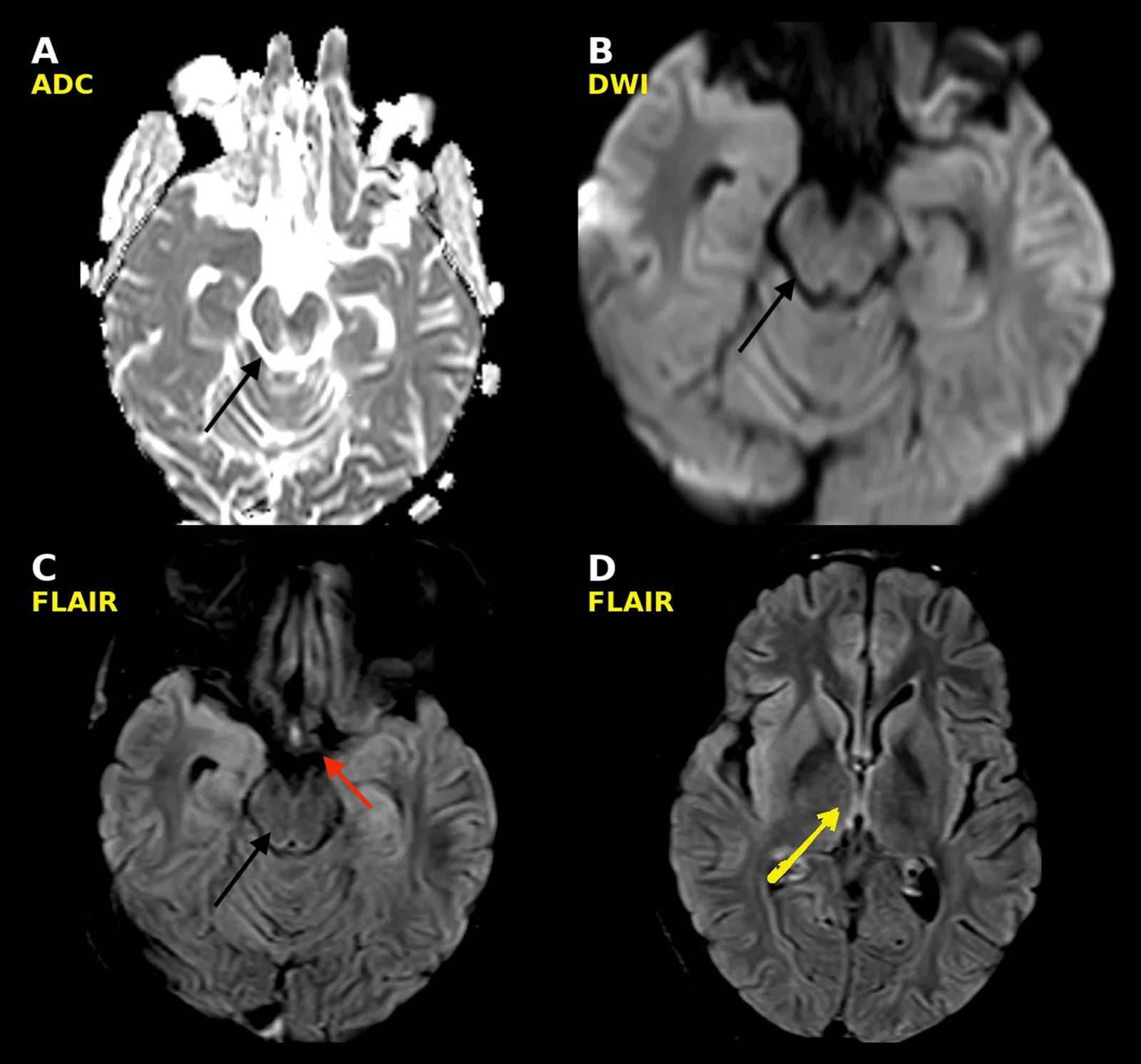
Brain magnetic resonance imaging (MRI) obtained during admission. (A) Apparent diffusion coefficient (ADC) map; the black arrow indicates the periaqueductal gray. (B) Diffusion-weighted imaging (DWI); the black arrow indicates the periaqueductal gray. (C) Axial T2 fluid-attenuated inversion recovery (FLAIR) at the level of the midbrain; the black arrow indicates the periaqueductal gray, and the red arrow indicates the mammillary body region. (D) Axial T2-FLAIR at the level of the third ventricle; the yellow arrow indicates the medial thalamus. The arrows indicate regions in which characteristic signal abnormalities may be seen in Wernicke encephalopathy (WE); no abnormal signal was identified in these regions in this patient.

Despite normalization of sodium and transaminases by the morning of day 4, the patient still showed little cognitive improvement and remained bedbound. Upon evaluation by our pediatrics team later that day, the patient was noted to have new-onset horizontal nystagmus with photophobia. The mother had also noticed an atypical gait while the patient was out of bed and walking to the bathroom. Neurological examination demonstrated truncal ataxia with an unstable gait but was otherwise without significant focal abnormalities. The patient's mother emphasized that these cognitive and neurological findings were atypical for his baseline demeanor and activity level. Physical and occupational therapy was subsequently consulted because of his prolonged inactivity and gait instability. Their evaluation confirmed truncal ataxia and mild right-sided dysmetria. A brain MRI was also ordered due to low cognitive improvement, new neurologic symptoms, and previous concern for SIADH. MRI showed “no pathologic intracranial enhancement,” with an impression of “normal MRI of the brain” (Figure 1).

Given the clinical constellation of nystagmus, ataxia, altered mentation, and a history of continued vomiting with significant weight loss in a patient, there was suspicion of WE. A thiamine blood test would be obtained but would take several days to return, as it needed to be sent to an outside laboratory. Considering the expected turnaround time for serum thiamine, the low risk of thiamine administration, and strong clinical suspicion for WE, treatment was initiated empirically after the initial thiamine level was collected. Neurology was subsequently consulted given our suspicion for WE and the neurological symptoms present in the patient. Ophthalmology was also consulted given the concerns for nystagmus.

Neurology reviewed the brain MRI that was obtained and agreed there were no findings to suggest an intracranial process as a reason for his neurological symptoms (Figure 1). They also agreed that hyponatremia was likely not the cause of his confusion given the level had normalized. Neurology agreed with our suggestion of empirically starting thiamine since vomiting is a possible etiology for vitamin deficiency. The patient was subsequently started on IV thiamine 500 mg every eight hours for two days and a multivitamin, followed by PO thiamine 100 mg for an additional four days. Neurology also recommended a full workup for encephalopathy, a one-hour EEG, and to continue monitoring electrolytes. The encephalopathy workup and EEG were both negative for any abnormal findings, and electrolytes had remained stable.

Within one day of thiamine supplementation (day 5 of admission), the patient's nystagmus and cognitive function improved. Ophthalmology saw the patient and agreed that nystagmus was not present and there were no concerns of impaired vision. The temporal correlation between thiamine supplementation and clinical improvement was consistent with a likely diagnosis of WE, and this was likely secondary to thiamine deficiency in the setting of prolonged emesis. On day 6 of admission, the patient’s truncal ataxia had improved, and he was able to ambulate with no assistance. On day 7, the thiamine test ordered previously confirmed a deficiency in vitamin B1 (thiamine) with a level of 39 nmol/L (Table 1). Given his clinical improvement and his B1 deficiency being treated, he was discharged with oral antiemetics and a daily multivitamin with planned outpatient follow-up. The patient agreed to stop cannabis use following discharge counseling. At his most recent follow-up with his primary care physician, the patient demonstrated adequate weight gain, full neurologic recovery, and a return to baseline activity. He also stated that he had no more episodes of emesis following cannabis cessation, supporting a diagnosis of cannabinoid hyperemesis syndrome as a likely cause of his vomiting and eventual vitamin deficiency.

## Discussion

To our knowledge, this represents one of the earliest reported pediatric cases of WE associated with CHS and the first reported pediatric case with negative neuroimaging [7]. Cannabinoid hyperemesis syndrome is a clinical diagnosis defined by episodes of emesis following chronic cannabis use and resolution of emesis following cannabis cessation. [2,3]. Its prevalence has increased alongside greater cannabis availability and increasing THC product potency. In our patient, emesis resolved following cannabis cessation, further supporting CHS as the likely cause of his prolonged vomiting.

Prolonged vomiting associated with CHS can result in reduced nutritional intake and volume depletion, with thiamine stores potentially becoming depleted within two to three weeks [3,7,8]. Thiamine functions as an essential cofactor for pyruvate dehydrogenase and other enzymes of carbohydrate metabolism. When thiamine is depleted, metabolism is unable to occur effectively, and lactic acid is produced, leading to the common neurological symptoms of WE [9]. Prolonged vomiting and volume depletion may also produce electrolyte and metabolic abnormalities that can cause neurologic manifestations overlapping with those of WE. Other more common etiologies of WE include hyperemesis gravidarum and severe eating disorders.

In our case, the patient demonstrated cognitive decline on initial evaluation. He presented with hyponatremia, transaminitis, substantial weight loss, and progressive nutritional depletion, which could have contributed to his neurologic decline. A multidisciplinary approach was used to manage these abnormalities. However, despite multiple consultations and subsequent normalization of his labs, there was little cognitive improvement, and nystagmus and truncal ataxia later developed. The encephalopathy workup, including brain MRI, was unremarkable, and suspicion for WE rested entirely on the typical triad of clinical symptoms. Rapid improvement occurred only after thiamine administration, supporting WE as the likely cause of his neurologic symptoms due to his frequent emesis episodes.

To our knowledge, Munroe et al. reported the other prior pediatric case of Wernicke encephalopathy associated with CHS [7]. Their patient was a 16-year-old male with chronic cannabis use who returned two months after a CHS diagnosis with dysarthria, dysmetria, ataxia, and altered mental status and with MRI findings typical of WE. The patient later improved rapidly on intravenous thiamine in the absence of any confirmatory thiamine lab value. In the adult population, Chaudhari et al. described a 41-year-old male who presented in status epilepticus from severe vomiting and hyponatremia, with bilateral thalamic T2/FLAIR hyperintensities on brain MRI and gradual improvement on intravenous thiamine [10]. Kattamuri et al. described a young woman with eight weeks of intractable vomiting and weight loss, bilateral lateral rectus palsy, horizontal nystagmus, and ataxic gait, with symmetric thalamic MRI hyperintensities and dramatic recovery following intravenous thiamine [11]. In contrast to all three prior reports, our patient's brain MRI demonstrated no acute findings, which could have led to a delay in treatment had the typical imaging pattern been required for diagnosis (Figure 1).

A central teaching point of this case is the limited sensitivity of neuroimaging for the diagnosis of WE, particularly in nonalcoholic and pediatric patients. Recent meta-analytic data report that classic T2/FLAIR hyperintensities in the mammillary bodies, periaqueductal grey, and thalami are present in only approximately 58% of patients with WE, with a pooled sensitivity of 53% and specificity of 93% [12]. In a 30-year pediatric WE systematic review, MRI sensitivity was higher at 85%, but the cohort excluded cases lacking confirmatory imaging, which introduces selection bias toward imaging-positive presentations [6]. The Caine operational criteria of dietary deficiency, oculomotor abnormalities, cerebellar dysfunction, and altered mental state or memory yield 85% sensitivity for autopsy-confirmed WE when at least two of four are met [13]. The European Federation of Neurological Societies guidelines explicitly state that imaging is supportive rather than required for diagnosis and recommend empiric high-dose intravenous thiamine in any suspected case prior to carbohydrate administration [14]. Our patient met four of the four Caine criteria (dietary deficiency, oculomotor abnormalities, cerebellar dysfunction, and altered memory state or memory) at the time of treatment initiation and demonstrated a clinically appropriate response to supplementation. A normal MRI should therefore not exclude the diagnosis of WE, particularly in a pediatric patient with a plausible etiology of thiamine depletion.

The multi-system course of prolonged emesis required multidisciplinary management of electrolyte, hepatic, and nutritional complications, but definitive treatment of suspected CHS remains cannabis cessation, which did occur in our patient [2,4]. For acute symptom control of CHS, the randomized Haloperidol Versus Ondansetron for Cannabis Hyperemesis Syndrome (HaVOC) trial demonstrated superiority of intravenous haloperidol over ondansetron for nausea and pain in adults [15], and a pilot randomized trial supports topical capsaicin to the abdomen as an adjunct [16]. Conventional antiemetics such as ondansetron and metoclopramide are of limited efficacy in CHS [8,17], which can prolong the symptomatic course. In our patient, empiric intravenous thiamine produced rapid clinical improvement, supporting a diagnosis of WE that was likely secondary to nutritional depletion in the setting of CHS.

Clinicians should consider WE when neurologic deficits persist despite correction of electrolyte and metabolic abnormalities. Delayed treatment risks progression to irreversible Korsakoff syndrome; therefore, the threshold for empiric thiamine should be low, and treatment should not be withheld while awaiting confirmatory laboratory values. A multidisciplinary approach is still recommended in managing the many abnormalities of prolonged vomiting and dehydrated states, but other diagnoses should be considered, particularly when neurologic symptoms fail to improve as expected, or new findings emerge.

## Conclusions

To our knowledge, this represents one of the earliest reported pediatric cases of Wernicke encephalopathy associated with CHS and highlights that normal MRI findings do not exclude the diagnosis. In chronic cannabis users with prolonged emesis, clinicians should consider cannabinoid hyperemesis syndrome in the differential diagnosis and remain alert to WE if neurologic symptoms develop. When WE is clinically suspected in the setting of prolonged CHS, empiric thiamine therapy should not be delayed while awaiting confirmatory laboratory or imaging findings.
